# Adhesive hydrogel delivering ALA prevents the malignant transformation of oral leukoplakia

**DOI:** 10.1016/j.mtbio.2026.103017

**Published:** 2026-03-10

**Authors:** Lin Lin, Jianchuan Ran, Yan Zhang, Shilin Guo, Xiteng Yin, Chuanchao Tang, Yufeng Wang, Wei Han, Wenmei Wang, Chuanhui Song

**Affiliations:** Nanjing Stomatological Hospital, Affiliated Hospital of Medical School, Institute of Stomatology, Nanjing University, Nanjing, 210008, China

**Keywords:** Hydrogel, Photodynamic therapy, Oral leukoplakia, Drug delivery, 5-Aminolevulinic acid

## Abstract

Photodynamic therapy (PDT) has shown promising efficacy for oral leukoplakia (OLK); however, its effectiveness is often compromised by the dynamic and wet oral environment, which impedes drug retention and complicates the maintenance of therapeutic concentrations. To overcome these limitations, we developed a mucoadhesive hydrogel composed of acrylic acid (AA), chitosan (CHI), and polydopamine for the controlled delivery of 5-aminolevulinic acid (ALA). The resulting ALA-loaded hydrogel (ALA-Gel) was synthesized via chemical cross-linking and demonstrated excellent wet adhesion capable of withstanding salivary flow, alongside sustained ALA release profiles. In vitro, ALA-Gel-mediated PDT markedly enhanced reactive oxygen species (ROS) generation and induced potent cytotoxicity in leukoplakia cells, while maintaining high biocompatibility with normal human keratinocytes. In a rat OLK model, the hydrogel achieved prolonged intraoral retention. Upon laser irradiation (632 nm, 100 mW cm^−2^, 100 J cm^−2^), both clinical and histopathological assessments confirmed a significant suppression of malignant progression. Furthermore, preliminary clinical trials (n = 30) demonstrated superior therapeutic outcomes compared to conventional treatments. Collectively, this hydrogel-based strategy addresses key challenges in oral PDT, offering a viable and translatable option for OLK management.

## Highlights

• An adhesive hydrogel for sustained 5-aminolevulinic acid delivery in oral leukoplakia PDT.
• Hydrogel ensures wet adhesion, efficient ROS, and superior cell kill in vitro and in vivo.
• Clinical trial confirms enhanced efficacy and comfort over conventional PDT for oral cancer prevention.

## Graphical abstract

**Figure:**
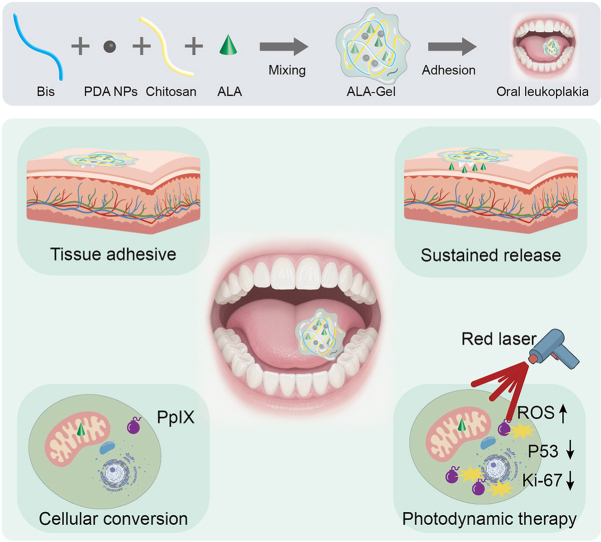

## 1 Introduction

Oral leukoplakia (OLK) is a well-established precancerous lesion characterized by white plaques on the oral mucosa, which can progress to oral squamous cell carcinoma without intervention [[1], [2], [3]]. Notably, OLK exhibits significant malignant potential, with epidemiological studies reporting an overall malignant transformation rate of 9.8% [4]. Effective management of OLK is therefore crucial for cancer prevention [5]. Conventional therapies, including surgery and pharmacotherapy, are often associated with high recurrence and malignant conversion rates [6,7]. As a promising alternative, 5-aminolevulinic acid (ALA)-based photodynamic therapy (PDT) is a minimally invasive modality with high selectivity for OLK management [8,9]. However, its clinical translation faces multiple challenges. First, topical ALA is used off-label for oral lesions in major markets such as the USA, Europe, and China, where no mucoadhesive formulation is commercially available for this indication [10,11]. Second, existing topical preparations require complex and inconvenient administration regimens (Fig. S1), significantly reducing patient compliance [12,13]. Finally, these formulations are poorly suited to the wet and dynamic oral environment, failing to maintain effective adhesion and sustained drug release, which results in low drug retention and bioavailability at the lesion site [14]. Although novel delivery systems (e.g., hydrogels, microneedles) have been investigated, a clinically viable, convenient adhesive patch for intraoral PDT remains unavailable [13,15]. Therefore, developing an intraoral adhesive drug delivery system is crucial to realizing the therapeutic potential of ALA-PDT.

**Fig. 1:**
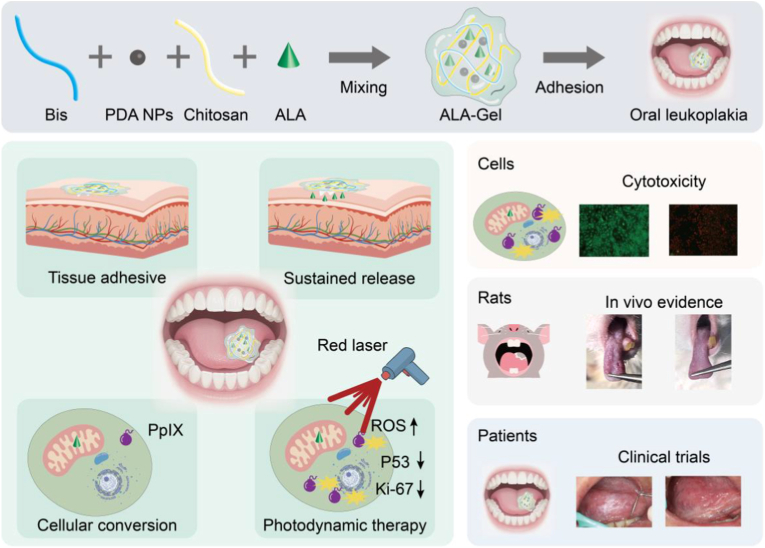
The synthesis and application of the ALA-Gel. The ALA-Gel was composed by the chitosan, acrylic acid, PDA and ALA via chemical cross-linking at suitable temperature. Upon application, the ALA-Gel adheres to the OLK lesion and rapidly releases the encapsulated ALA. The released ALA is taken up by cells and metabolized into PpIX. Subsequent laser irradiation activates PpIX, generating ROS that eliminate abnormal cells and suppress malignant transformation.

To address these limitations, we engineered a mucoadhesive ALA-loaded hydrogel (ALA-Gel) to prevent OLK malignant transformation (Fig. 1). Hydrogels have attracted significant scientific interest due to their exceptional retention capabilities, adhesive properties, and biocompatibility [[16], [17], [18], [19], [20], [21]]. Drug-encapsulating hydrogels can increase effective drug concentrations at lesion sites, thereby enhancing therapeutic efficacy [[22], [23], [24], [25], [26], [27], [28]]. These characteristics render hydrogels a transformative platform for targeted and sustained drug delivery [[29], [30], [31]]. Incorporating polydopamine (PDA) as a functional agent enhances the hydrogel's wet-adhesion properties, making it suitable for oral cavity applications [[32], [33], [34]]. Additionally, ALA is a widely utilized precursor of the photosensitizer protoporphyrin IX (PpIX), which generates high levels of reactive oxygen species (ROS) upon laser irradiation [35]. PDT is a highly selective, minimally invasive treatment with established clinical utility for malignant and premalignant lesions [36]. ALA-PDT has advanced applications in dermatology, superficial tumor therapy, and oral oncology [37,38], with well-documented biosafety and efficacy in both preclinical and clinical settings [39]. Thus, ALA delivery via adhesive hydrogels represents a promising therapeutic strategy for OLK.

In this study, we developed a mucoadhesive hydrogel system comprising acrylic acid (AA), chitosan (CHI), and PDA for ALA delivery to treat OLK. Chemical cross-linking of these components produced a homogeneous ALA-Gel with robust adhesive properties, enabling firm adhesion to moist tissues. The encapsulated ALA demonstrated sustained in situ release, maintaining elevated local drug concentrations at lesion sites. Following ALA pretreatment, laser irradiation triggered a marked increase in intracellular ROS levels, leading to cell death. The ALA-Gel demonstrated prolonged adhesion to tongue lesions in both OLK rat models and human patients. Combined with PDT, this strategy suppressed OLK malignant transformation, as evidenced by reduced Ki-67 and p53 expression. These findings indicate that ALA-Gel effectively inhibits malignant transformation progression at lesion sites, representing a promising minimally invasive in situ strategy for OLK treatment.

## 2 Results and discussion

### 2.1 Construction and characterization of ALA-gel

**Fig. 2:**
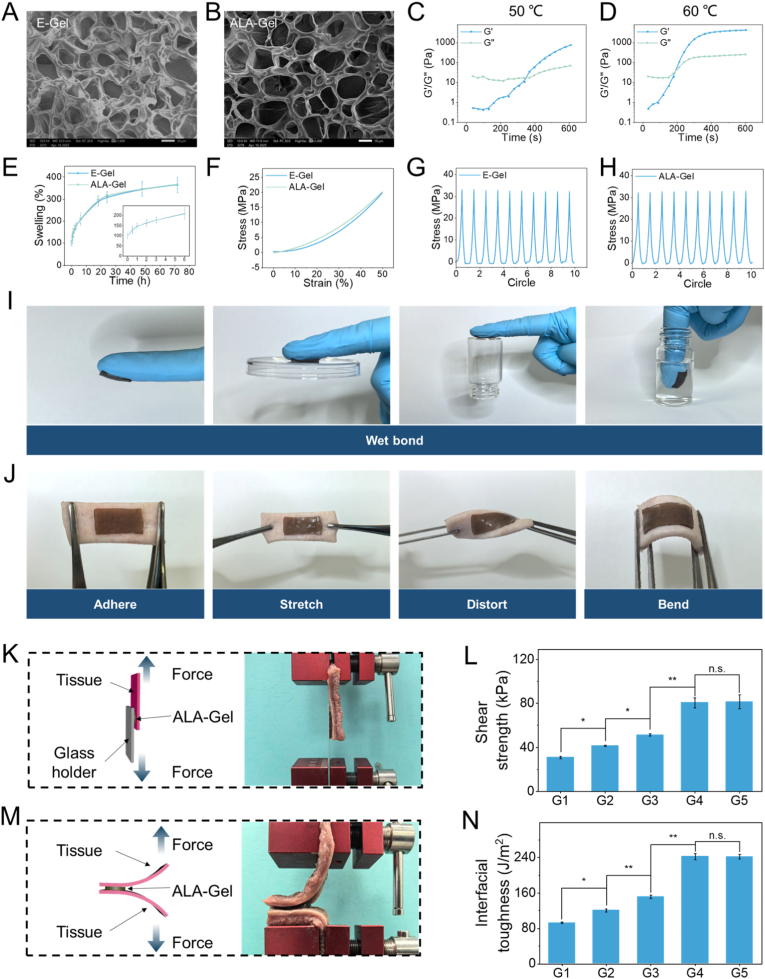
Physical characterization of the hydrogels. (A, B) The SEM images of the E-Gel and ALA-Gel. Scale bar = 10 μm. (C, D) Time-sweep showing the evolution of the storage modulus (G′) and loss modulus (G″) for the hydrogel. Measured with an oscillation frequency of 0.1 Hz at 50 °C and 60 °C. (E) Swelling ratios of the E-Gel and ALA-Gel. (F) Compressive stress-strain curves of different hydrogel formulations. (G, H) Cyclic tensile stress-strain curves of the hydrogel under successive loading cycles. (I) Adhesive performance of the ALA-Gel on various substrates. (J) Ex vivo adhesion of the ALA-Gel on porcine skin. Determination of adhesive abilities of hydrogel based on (K, L) lap shear test and (M, N) 180° peel test, respectively. (K, M) Scheme and optical images exhibiting the adhesive type and process of hydrogel on the tissue; (L, N) maximum shearing strengths and interfacial toughness. E-Gel: empty hydrogel; ALA-Gel: hydrogel with ALA; G1: CHI-only; G2: AA-only; G3: E-gel (without PDA); G4: E-gel; G5: ALA-gel.

ALA-Gel was constructed from four primary components: AA, CHI, PDA, and ALA, with N, N′-methylenebisacrylamide (BIS) serving as the cross-linker to enhance structural stability. AA serves as the primary monomer, forming a polyacrylic acid network via polymerization to constitute the hydrogel matrix. CHI, a cationic polysaccharide derivative, was incorporated to enhance biocompatibility and interact with ALA [13]. PDA was introduced to provide adhesion through covalent and hydrogen bonding with biological tissues [40]. Microstructural analysis revealed no significant alterations following ALA loading, comparing the Empty-load hydrogel (E-Gel, empty hydrogel) and the ALA-Gel (Fig. 2A and B). Oscillatory rheometry was employed to characterize ALA-Gel's rheological properties. As shown in Fig. 2C and D, temperature influences hydrogel crosslinking kinetics, with accelerated formation at elevated temperatures (60 °C v.s. 50 °C). ALA incorporation exerted minimal effects on hydrogel swelling behavior (Fig. 2E). Stress-compression curves showed negligible differences pre- and post-swelling (Fig. S2A). Tensile strain remained comparable following ALA addition (Fig. 2F). Ten cyclic tensile tests confirmed ALA-Gel's superior fatigue resistance (Fig. 2G–H). Adhesive effectiveness was evaluated using latex gloves to simulate adhesion to plastics, glass, and underwater substrates (Fig. 2I). Ex vivo porcine skin served as a model tissue for adhesion assessment, with the hydrogel maintaining firm attachment during mechanical stress (Fig. 2J). To confirm the adhesive properties of the prepared hydrogel, we conducted standardized lap-shear and 180° peel tests on freshly excised porcine tongues to quantitatively assess adhesive strength. ALA-Gel exhibited a shear strength of 81.4 ± 6.4 kPa, which was similar to that of E-Gel (80.5 ± 4.5 kPa) and higher than E-Gel (without PDA) (51.3 ± 0.9 kPa) (Fig. 2K and L). In addition, ALA-Gel showed an adhesion energy of 242.1 ± 5.3 J m^−2^, which was higher than that of CHI-only (92.5 ± 1.9 J m^−2^), AA-only (120.4 ± 3.5 J m^−2^) and E-gel (without PDA) (150.9 ± 4.0 J m^−2^) in the 180-degree peel test, (Fig. 2M and N). Collectively, these data demonstrate ALA-Gel's suitability for oral cavity application.

### 2.2 ALA-gel enhancing PDT efficiency

**Fig. 3:**
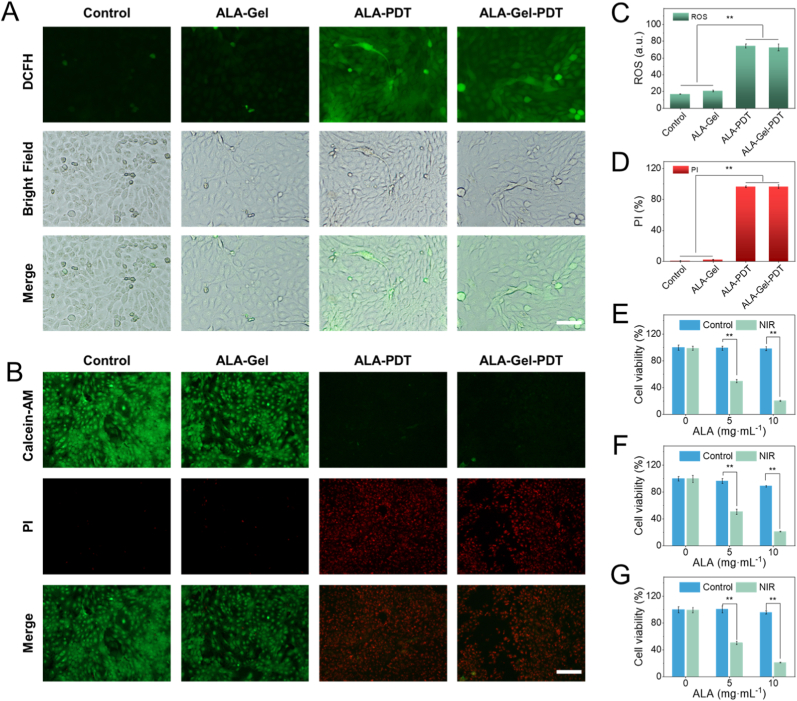
In vitro cell cytotoxic effects of the ALA-Gel. (A) The DCFH-DA staining of the Leuk-1 cells showing the cellular ROS level. (B) Viability of Leuk-1 cells after various treatments assessed by Calcein-AM/PI staining (live cells: green; dead cells: red). (C) The quantitative data of the ROS level. (D) Quantification of cell death rates. Cell viability of (E) Leuk-1, (F) DOK, and (G) CAL-27 with different concentrations of the ALA. Scale bar = 100 μm. (For interpretation of the references to colour in this figure legend, the reader is referred to the Web version of this article.)

To examine the ALA-Gel's effectiveness, we compared the photocytotoxic effects of free ALA and ALA-Gel in dysplastic oral keratinocyte cells (Leuk-1). ALA is metabolized to PpIX inside cells. Upon laser irradiation, PpIX produces excessive ROS to destroy the malignant cells, which is widely applied in the clinic. To investigate the spatiotemporal dynamics of drug release of the ALA-Gel, we developed an in vitro model via using a vertical Franz diffusion cell to mimic the time-related release process of ALA. The result showed that the inner ALA was rapidly released within the first 30 min (Fig. S2B). The DCFH was used to evaluate oxidative stress, showing green fluorescence under fluorescent microscopy. After the ALA and ALA-Gel were applied, the cells had high cellular ROS levels, confirming their photodynamic therapy potential (Fig. 3A). Quantitative data showed that the cells in the ALA and ALA-Gel groups had significantly high levels of ROS compared with the control group (Fig. 3C). To further evaluate the cytotoxic activity against malignant cells by the Calcein-AM/PI staining, which can distinguish the living/dead cells with green/red fluorescence, respectively. Consistent with the ROS level, the ratio of the dead cells was also high in the ALA and ALA-Gel based PDT (Fig. 3 B and D). We also assessed the photocytotoxic effects of ALA-Gel on Leuk-1 (Fig. 3 E), DOK (dysplastic oral keratinocyte) (Fig. 3F) and CAL-27 (oral squamous cell carcinoma) (Fig. 3G) cell lines. The cell count kits showed that the ALA-Gel-PDT could also destroy the cells effectively, broadening the application scenarios of the prepared drug delivery hydrogel.

### 2.3 ALA-gel has its own bio-safety on the normal cells

**Fig. 4:**
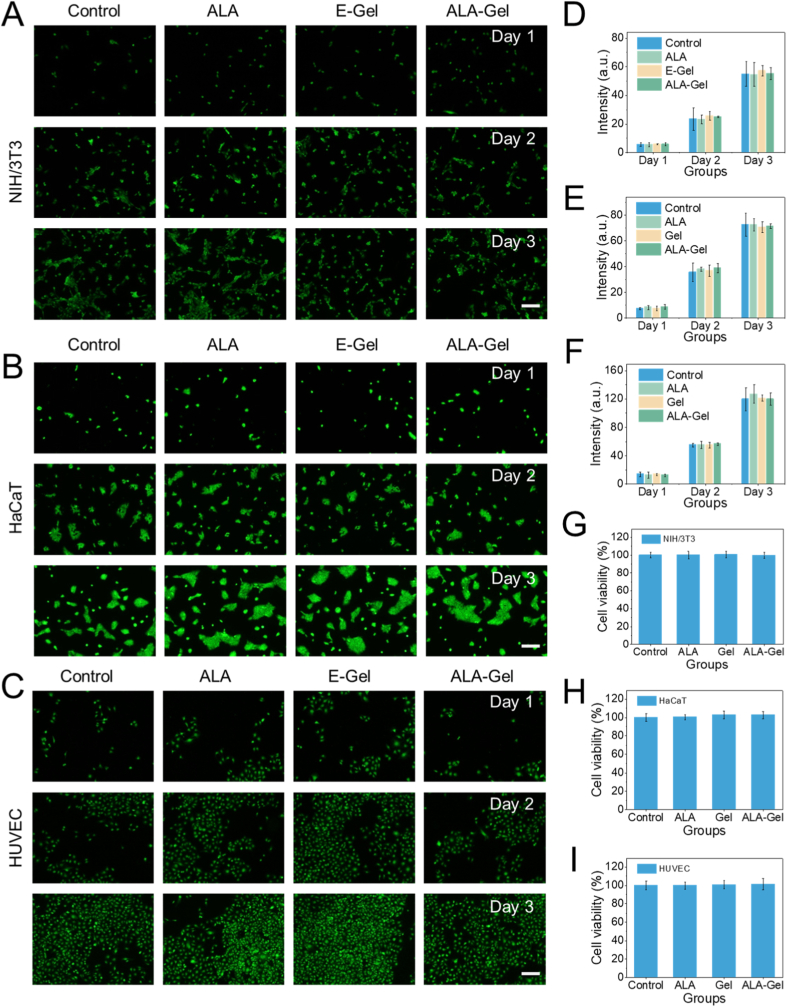
Biosafety evaluation of ALA-Gel. (A–C) Cell viability of (A) NIH/3T3, (B) HaCaT, and (C) HUVEC cells co-cultured with different hydrogel formulations, assessed by Calcein-AM/PI staining (live cells: green; dead cells: red). (D–F) Quantification of cell viability for (D) NIH/3T3, (E) HaCaT, and (F) HUVEC cells. (G–I) Cell proliferation of (G) NIH/3T3, (H) HaCaT, and (I) HUVEC cells treated with different groups, determined by CCK-8 assay. (For interpretation of the references to colour in this figure legend, the reader is referred to the Web version of this article.)

Given direct tissue contact during application, a biosafety assessment was imperative. To investigate the biosafety of the ALA-Gel, we used mouse fibroblast cells (NIH/3T3), human keratinocyte line (HaCaT), and human endothelial cell line (HUVEC) to culture with the gel [41]. After three days, the calcein-AM/PI staining was used to show the cells' growth situation. The green fluorescence revealed that the 3T3 cells were increasing gradually, indicating that the cells were in a normal state. And the quantitative data also showed no apparent difference between groups (Fig. 4A and D). Also, the results of the other two cells were similar (Fig. 4B, C, E, and F). The CCK-8 test also showed that the hydrogel without laser did not affect cell growth (Fig. 4G–I). All these data indicated that the prepared hydrogel has promising biosafety.

### 2.4 Therapeutic efficacy of ALA-gel in a carcinogenesis model

**Fig. 5:**
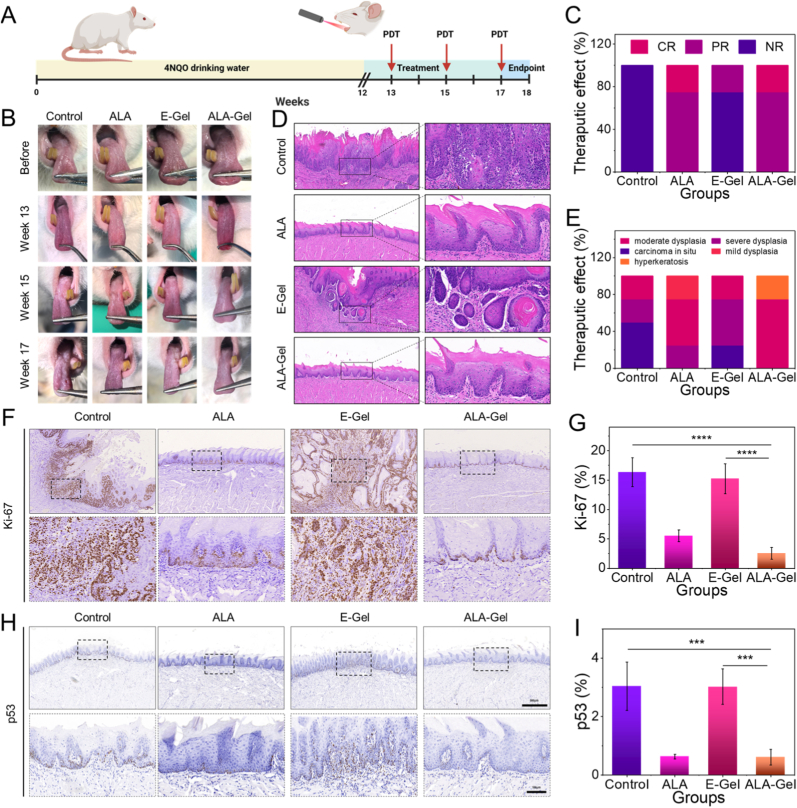
In vivo therapeutic efficacy of ALA-Gel in a rat OLK model. (A) Schematic diagram of the experimental procedures. (B) Representative clinical photographs of rat tongues during treatment. (C) Clinical evaluation of therapeutic effects. (D) Pathological staining of the rat tongue tissue after treatment and local magnified images. (E) Pathological evaluation of therapeutic effects. (F) Representative IHC staining of Ki-67 in tongue tissues. (G) Quantification of Ki-67-positive cells. (H) Representative IHC staining of p53 in tongue tissues. (I) Quantification of p53-positive cells.

Therapeutic efficacy was evaluated in a 4-nitroquinolin-1-oxide (4NQO)-induced rat OLK model [3]. Fig. 5A illustrates the specific time points for modeling. At week 12, the tongue tissues from two randomly selected rats were subjected to hematoxylin and eosin (H&E) staining, revealing the presence of oral leukoplakia, thereby confirming the successful establishment of the model (Fig. S3). The rats were randomly assigned to four groups: control, ALA, E-Gel, and ALA-Gel. Owing to its superior adhesive properties, the hydrogel remained firmly attached to the rat tongues for up to 3 h without dislodge during feeding (Fig. S4). Photodynamic therapy was administered to the rats at weeks 13, 15, and 17. Treatment efficacy was evaluated as detailed in the Methods section (Section 4.9). Following the 4-week intervention, the dorsal tongue surfaces were assessed, and the white lesion areas were quantified and subjected to statistically analyzed (Fig. 5B, C, and S4B). At the experimental endpoint (week 18), histological examination using H&E staining was performed to assess oral epithelial dysplasia (OED). The outcomes of the aforementioned groups above are illustrated in Fig. 5D and E, demonstrating that hydrogels containing ALA significantly reduced the occurrence of neoplasms. Immunohistochemical (IHC) analysis of Ki-67 expression demonstrated a considerable proliferation in the ALA-Gel group (2.54 ± 1.13% positive cells) compared to both the control (16.35 ± 2.81%, p < 0.0001) and E-Gel groups (15.25 ± 2.92%, p < 0.0001). Notably, the ALA-Gel group showed no significant difference in Ki-67 positivity compared with the ALA-alone group (p = 0.27). Consistent with the abovementioned findings, p53 IHC staining demonstrated similar patterns, indicating that the ALA-Gel effectively suppressed epithelial cell proliferation and potentially prevented the malignant transformation of OLK (Fig. 5F–I). Histopathological and biochemical analyses revealed no abnormalities in major organs or liver/kidney function, confirming the hydrogel's biosafety (Fig. S5).

### 2.5 Clinical adhesion assessment and efficacy evaluation of ALA-gel

**Fig. 6:**
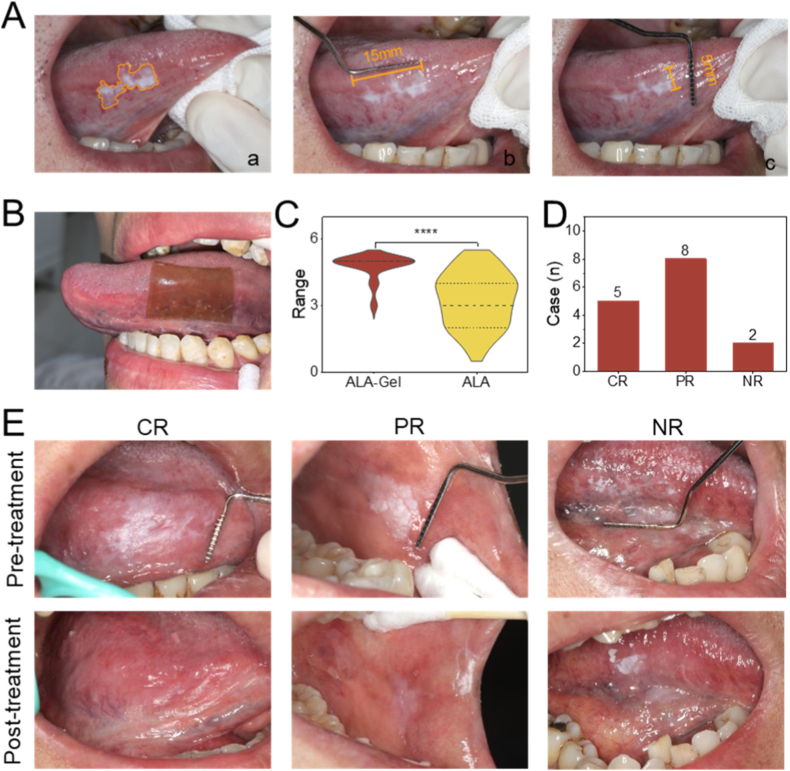
Clinical patient application evaluation of the ALA-Gel. (A) The quantitative diagram of the lesion area. (B) The clinical application scenarios in a patient's oral cavity. (C) The difference in lesion area between the two groups. (D) Evaluation of the clinical effects of the two groups of patients. (E) Representative images of the clinical effects of the two groups of patients.

To evaluate the clinical adhesion properties and patient comfort of ALA-Gel DDS, this trial enrolled 30 volunteers. A complete course of photodynamic therapy was administered to each patient, which was defined as continuing until either complete resolution of the lesion was achieved or the lesion area stabilized with no observable change. Each patient may present with one or more lesions, which are distributed across various regions of the oral cavity, including the cheek, lip, tongue, etc. The lesion area was quantified before and after the trial using a standardized periodontal probe and ImageJ software for two-dimensional analysis (Fig. 6A). The ALA-Gel DDS was administered to the mucosal surface under controlled moisture conditions and fixed in place with 5 s of gentle pressure (Fig. 6B). Notably, patients maintained normal speech during the procedure-a significant advantage over conventional dressings that typically require oral immobilization (Video S1). Adhesion performance was quantitatively assessed at 30, 60, and 120 min post-application, showing progressive displacement rates of 0%, 3%, and 10%, respectively. Three instances of displacement were observed at the study endpoint, including one case caused by involuntary swallowing reflexes and two cases resulting from hypersalivation. For subjective assessment of device comfort, participants completed a standardized 5-point Likert scale (1 = extremely uncomfortable; 5 = extremely comfortable), while simultaneously evaluating impacts on speech articulation and swallowing function. The cohort (n = 30) reported a mean comfort score of 4.77 (range: 3-5), demonstrating statistically significant improvement over conventional delivery systems (mean: 3.27; p < 0.001). These quantitative findings were supported by qualitative participant feedback, which highlighted excellent functional compatibility (Fig. 6C). Following 2-3 h of ALA-Gel DDS application, the residual hydrogel patches were removed. In fifteen subjects, fluorescence response was assessed under UV irradiation (405 nm), after which standard PDT was administered using a 632 nm laser system. Therapeutic outcomes were evaluated at 4-week follow-up. Clinical outcomes demonstrated CR in 5 patients (33.3%), PR in 8 patients (53.3%), and NR in 2 patients (13.3%), yielding an overall response rate of 86.7%. This represents a clinically meaningful improvement compared to conventional therapy [39] (77.4%, p = 0.032, Fig. 6D and E).

Supplementary data related to this article can be found online at https://doi.org/10.1016/j.mtbio.2026.103017

The following are the Supplementary data related to this article.

Video s1 Video s1

It is important to acknowledge the inherent trade-offs between topical and systemic ALA delivery in the context of PDT for oral lesions. Systemic administration, typically via an oral solution (e.g., Gleolan), has demonstrated excellent clinical efficacy, often achieving complete lesion clearance in a single treatment cycle due to deep and uniform protoporphyrin IX (PpIX) accumulation throughout the target tissue [42]. However, this route induces sustained systemic photosensitivity, typically lasting 24 to 48 h, necessitating stringent patient precautions and impacting quality of life. In contrast, topical administration aims to confine photosensitization locally, thereby avoiding systemic side effects. Yet, conventional topical formulations often struggle to penetrate the oral keratinized mucosa, frequently necessitating multiple treatment cycles to achieve a complete response. The present study addresses this specific limitation by developing an optimized topical formulation designed to enhance ALA penetration. Our approach seeks to bridge this clinical trade-off: by improving local bioavailability through topical delivery, we aim to reduce the treatment burden of repeated applications while potentially minimizing the risk of prolonged systemic photosensitivity associated with oral ALA administration. The ideal regimen would balance high single-session efficacy with a favorable localized safety profile, and our work represents a step toward this goal. Furthermore, the critical aspect of local off-target transfer risk was addressed through direct clinical observation and procedural precision. In vivo application confirmed that the hydrogel adheres robustly to the target lingual mucosa without noticeable detachment or migration into adjacent non-target tissues (e.g., cheek or gingiva) during the therapeutic window, as shown in Video S1. This inherent retention is synergistically reinforced by our spatially confined PDT protocol. The 632 nm laser spot was meticulously calibrated and delivered exclusively to the predefined lesion area, ensuring that the photoactivation of ALA—and thus the therapeutic effect—was strictly limited to the target pathology. This dual strategy of inherent material localization and precision activation establishes a reliable safety profile by fundamentally minimizing the potential for off-target effects.

## 3 Conclusion

In conclusion, we developed an adhesive hydrogel for ALA delivery to enhance PDT efficacy and prevent OLK malignant transformation. Although clinically established, conventional ALA-PDT involves complex procedures that cause patient discomfort. Despite significant efforts to improve drug delivery efficacy, inadequate adhesion and patient discomfort remain persistent challenges. We incorporated polydopamine to enhance hydrogel adhesive performance through its underwater adhesion capabilities, amplifying localized drug concentrations and optimizing patient comfort. ALA-Gel was synthesized via chemical cross-linking and exhibits excellent wet-adhesion properties suitable for oral cavity applications. Sustained ALA release occurred at lesion sites following application. In vitro studies confirmed its efficacy in generating ROS and inducing leukoplakic cell death. Coculture experiments demonstrated exceptional biocompatibility with normal cells. In rat OLK models, prolonged intraoral retention was observed. Laser irradiation effectively suppressed leukoplakic malignant transformation. Clinical application in patients significantly improved comfort levels and therapeutic outcomes versus conventional treatments. This strategy represents a viable clinical option for OLK management with substantial translational potential.

## 4 Methods

### 4.1 Reagents

Acrylic acid (AA), chitosan (CHI), dopamine, N, N′-methylenebisacrylamide, Tris, Ammonium persulfate, 5-aminolevulinic acid hydrochloride, cell culture medium, and PBS were purchased from Adamas-beta. The 4NQO (catalog N8141) was purchased from Sigma–Aldrich. Aminolevulinic acid hydrochloride for topical power (118 mg/vial) was purchased from Fudan Zojyo Biomed. A fresh 20% (w/v) aqueous solution was prepared for each experiment by dissolving the powder in sterile water for injection, which was then promptly mixed into the hydrogel carrier. Artificial Saliva was obtained from the Solarbio. Porcine tongue tissues were obtained from a local abattoir.

### 4.2 Preparation of ALA-gel

The PDA was synthesized according to the literature [43], and the brief steps are as follows: dopamine was added to the Tris-HCl buffer (pH = 8.5) at a concentration of mg/mL. The solution was stirred continuously for 18 h under dark conditions. After reaction, the PDA was centrifuged (30,000 g, 15 min), and lyophilized.

Briefly, a mixture of 1.5g AA, 0.15g CHI, and 0.05g PDA was dispersed to the container, and the mixture was dispersed into deionized water with continuous stirring. Subsequently, 4 mg of BIS was added as a cross-linker, and the mixture was stirred for 20 min to achieve complete dissolution. In a separate step, 0.04 g of ammonium persulfate (APS) and 0.15 g ALA were dissolved in a designated amount of distilled water. This solution was then added dropwise to the monomer mixture, stirred vigorously. The final water content of the entire pre-polymerization solution was adjusted to 77 wt%. The resulting mixture was sonicated to remove entrapped bubbles and subsequently polymerized in a water bath at 60 °C for 2 h. The resulting ALA-Gel contained ALA at a theoretical final concentration of 23.6 mg/mL within the aqueous phase. Cylindrical samples were prepared using molds with an inner diameter of 3 mm. The obtained hydrogels were sectioned into thin patches using a freezing microtome, dried, sealed in plastic bags with desiccant, and stored at 4 °C for future use.

### 4.3 Characterization of the ALA-gel

After cross-linking, the empty hydrogel and the hydrogel containing ALA were freeze-dried to obtain dry sheets. The SEM observed the sheets. The HAAKE™ MARS 40 was used to analyze the oscillatory rheological measurement under different temperatures. The freeze-dried E-Gel and ALA-Gel were put into the artificial salvia, and the hydrogel was measured at the selected time at 37 °C. The swelling ratio was calculated using the following formula:

Swellingratio(%)=(W2−W1)/W1×100%

W1: original weight; W2: weight after stay in the artificial salvia.

Tensile tests were carried out on a tensiometer equipped with a 10 N load cell at a 100 mm min^−1^ crosshead speed. The cylindrical samples tested were 10 mm long and had a gauge length of 5 mm. A maximum strain of 100% was chosen for the cyclic tensile tests.

To test the adhesive property, the ALA-Gel was attached to latex gloves, and then various materials, such as plastic petri dishes and glass sample bottles, were attached to the hydrogel. The fresh pork skin was obtained from the supermarket. The hydrogel was attached to the skin, and the adhesion situation was observed after different exercises.

The shear strength and 180° peel adhesion of the hydrogel on porcine lingual mucosa were measured using a mechanical testing machine. To simulate the oral environment, artificial saliva was applied to the mucosa prior to testing. For the shear strength test (lap-shear method), a rectangular mucosal specimen (25 mm × 60 mm) was prepared. The hydrogel was adhered to the mucosa and fixed to a glass slide on the opposite side. Shear strength was calculated as F_max_/adhesion area. For the 180° peel test, two mucosal specimens (25 mm × 60 mm) were used. The hydrogel was adhered between them over a 20-mm diameter interface, and the tissue ends were clamped.

### 4.4 The ALA release profile of the ALA-gel

Drug release was assessed using a vertical Franz diffusion cell (effective diffusion area: 0.63585 cm^2^). The hydrogel was applied on top of freshly excised porcine lingual mucosa as the barrier membrane. The receptor compartment was filled with artificial saliva (pH 6.8) and maintained at 37 °C with constant magnetic stirring at 300 rpm to ensure sink conditions throughout the experiment. Samples from the receptor were taken at predetermined time intervals and analyzed via UV-Vis spectroscopy at 275 nm using a validated calibration curve for ALA.

### 4.5 The ROS detection of the cells

The leuk-1 cells were cultured in the DMEM culture medium with 10% FBS at 37 °C. After the cells adhere to the surface, the ALA and the ALA-Gel are added. After coculture for 3h, a 632 nm laser was applied for 10 min. The intracellular reactive oxygen levels in the cells were examined using DCFH. The confocal laser scanning microscope (CLSM) detected the fluorescence of the cells. And ImageJ is used for quantitative analysis.

### 4.6 The living/dead staining of the cells

The leuk-1 cells were added to the 6-well plates, and the ALA and the ALA-Gel were added at the same drug concentration. The next day, the 632 nm laser was applied for 10 min per well, after which, the calcein-AM/PI (2μM/4 μM) was stained for 30 min to distinguish the living/dead cells. Then the CLSM and the ImageJ were utilized to observe and analyze the cells.

### 4.7 The cell toxicity of the ALA-gel

The Leuk-1, DOK, and CAL-27 cells were used as the target cells to evaluate the ALA-Gel-based PDT. The cells were seeded into the 96-well cell plate. After different doses of ALA-Gel were added, the cells were cultured 3h. The 632 nm laser was used for PDT. After 10 min of irradiation, the CCK-8 cell count kit was added at 10% concentration to analyze the cell number.

### 4.8 The biosafety of the ALA-gel

The NIH/3T3, HUVEC and HaCaT cells were cultured in the DMEM culture medium. The ALA, E-Gel and ALA-Gel were added to the cell culture. The cells were cultured for three days, and calcein (2 μm) was added for staining the living cells.

### 4.9 Light source and standard PDT procedure

PDT was performed using an LH-400 medical diode laser (Tianjin Leiyi Laser Technology Co., Ltd., China) with a central wavelength of 635 nm. The laser output was coupled into a sterile, multimode optical fiber (0.6-0.8 mm core diameter) terminated with a microlens. The fiber was held perpendicular to the lesion surface at a fixed working distance to ensure a consistent and uniform circular irradiation spot with a diameter of 1.0 cm. The output power was set to 100 mW and verified using with a calibrated FieldBest-Il-6W laser power meter (Xunmiao Optoelectronics Co., Ltd.) prior to each treatment session. The irradiance (power density, P) at the tissue surface was calculated using the formula ρ = 4 Pt/πd^2^, where ρ is the total fluence (100 J cm^−2^), t is the total irradiation time, and d is the spot diameter (1.0 cm). A fractionated irradiation protocol was employed, consisting of 3-min light cycles alternated with 3-min dark intervals. The number of cycles was determined according to the lesion volume to achieve the target total fluence of 100 J cm^−2^. The irradiation spot was positioned to extend 2-3 mm beyond the lesion margins. For larger lesions, adjacent spots were systematically overlapped to guarantee complete and homogeneous coverage [11].

### 4.10 Treatment endpoint and efficacy evaluation

Each patient received a complete course of photodynamic therapy, with the treatment endpoint defined as the point at which either complete resolution of the lesion was observed or the lesion area stabilized (i.e., showed no observable change over time). Therapeutic efficacy was assessed based on standardized criteria as follows: complete response (CR) was defined as the total disappearance of the lesion; partial response (PR) was defined as a reduction in lesion area of ≥30%; and no response (NR) was defined as a reduction of <30% or disease progression. The overall treatment efficacy (TR) was subsequently calculated as the proportion of patients achieving either CR or PR, using the formula: TR = (CR + PR)/(CR + PR + NR) × 100% [9].

### 4.11 4NQO-induced rat OLK model

All animal experiments were conducted following NIH guidelines and approved by the Nanjing University Ethics Committee (Approval No. IACUC-D2402059). Six-week-old Wistar rats were acclimatized for one week under standard housing conditions before initiating the 4NQO-induced OLK model. Animals were administered 4NQO (100 μg mL^−1^) in their drinking water for 12 weeks while maintaining a standard diet. Following successful OLK induction, rats were randomly divided into four groups (n = 4): 1) Control group receiving sterile water injection; 2) ALA group receiving local injection of 100 μL 20% ALA solution; 3) E-Gel group treated with blank hydrogel; and 4) ALA-Gel group receiving ALA-loaded hydrogel. After 3h drug incubation, PDT was then performed according to the procedure outlined in *Methods 4.8*. The four-week treatment period allowed unrestricted access to food and water, with weekly tongue examinations. At the experimental endpoint (week 18), rats were euthanized for tissue collection. Tongue specimens and major organs were paraffin-embedded and processed for H&E, Ki-67, and p53 immunohistochemical staining. OED grading was performed independently by three blinded pathologists. Blood samples were analyzed for biochemical parameters.

### 4.12 Clinical data of the patients

This study included thirty patients with OLK who underwent treatment at the Department of Oral Mucosal Diseases, Nanjing Stomatology Hospital from July 2023 to July 2024. The patient cohort, comprising 18 males and 12 females (3:2 ratio), had a mean age of 51.8 ± 16.6 years (range: 24–79). Each patient underwent a minimum of one ALA-PDT session. This study was approved by the Ethics Committee of Nanjing Stomatological Hospital, Medical School of Nanjing University (Approval No. NJSH-2023NL-093-1). Written informed consent was obtained from all participants included in the study prior to their enrollment. Detailed inclusion and exclusion criteria are provided in Supplementary Table 1. Patient allergy histories were recorded, and standardized clinical photographs were taken at baseline and throughout the follow-up period. No participant withdrawals occurred.

### 4.13 Statistical analysis

Origin software was used to calculate the p-value using the Student's t-test to compare differences between two groups or one-way analysis of variance (ANOVA), followed by Turkey's multiple comparisons and two-way ANOVA, followed by Turkey's multiple comparisons to compare differences between more than two groups. In all cases, statistical differences were considered at ∗p < 0.05, ∗∗p < 0.01, ∗∗∗p < 0.001, and ∗∗∗∗p < 0.0001; not significant (ns) was assigned when p ≥ 0.05.

## CRediT authorship contribution statement

**Lin Lin:** Conceptualization, Data curation, Funding acquisition, Resources, Validation, Visualization, Writing – original draft. **Jianchuan Ran:** Data curation, Formal analysis, Investigation. **Yan Zhang:** Conceptualization, Data curation, Investigation. **Shilin Guo:** Formal analysis, Funding acquisition, Supervision. **Xiteng Yin:** Conceptualization, Data curation, Funding acquisition. **Chuanchao Tang:** Resources, Software, Supervision. **Yufeng Wang:** Funding acquisition, Software. **Wei Han:** Conceptualization, Funding acquisition, Investigation, Resources, Supervision, Writing – review & editing. **Wenmei Wang:** Conceptualization, Investigation, Methodology, Supervision. **Chuanhui Song:** Conceptualization, Data curation, Funding acquisition, Supervision, Writing – review & editing.

## Declaration of competing interest

The authors declare that they have no known competing financial interests or personal relationships that could have appeared to influence the work reported in this paper.

## Data Availability

Data will be made available on request.

## References

[1] Sung H., Ferlay J., Siegel R.L. (2021). Global cancer statistics 2020: GLOBOCAN estimates of incidence and mortality worldwide for 36 cancers in 185 countries. CA Cancer J. Clin..

[2] Cunha A.R.D., Compton K., Xu R. (2023). The global, regional, and national burden of adult lip, oral, and pharyngeal cancer in 204 countries and territories: a systematic analysis for the global burden of disease study 2019. JAMA Oncol..

[3] Lin L., Song C., Wei Z. (2022). Multifunctional photodynamic/photothermal nano-agents for the treatment of oral leukoplakia. J. Nanobiotechnol..

[4] Du Y., Liu T., Ding T. (2024). Adhesive lipophilic gels delivering rapamycin prevent oral leukoplakia from malignant transformation. Mater. Today Bio.

[5] Chaturvedi A.K., Udaltsova N., Engels E.A. (2020). Oral leukoplakia and risk of progression to oral cancer: a population-based cohort study. J. Natl. Cancer Inst..

[6] Kawczyk-Krupka A., Waskowska J., Raczkowska-Siostrzonek A. (2012). Comparison of cryotherapy and photodynamic therapy in treatment of oral leukoplakia. Photodiagnosis Photodyn. Ther..

[7] Hanna G.J., Villa A., Nandi S.P. (2024). Nivolumab for patients with high-risk oral leukoplakia: a nonrandomized controlled trial. JAMA Oncol..

[8] Xu T., Zhong L., Liu Q. (2025). NRF2 modulates WNT signaling pathway to enhance photodynamic therapy resistance in oral leukoplakia. EMBO Mol. Med..

[9] Song Y., Tang F., Liu J. (2024). A complete course of photodynamic therapy reduced the risk of malignant transformation of oral leukoplakia. Photodiagnosis Photodyn. Ther..

[10] Li D., Yu Z., Nie C. (2023). Analysis of drug factors in 5-aminolevulinic acid photodynamic therapy for oral potentially malignant disorder. West China J. Stomatol..

[11] Chen Q., Dan H., Tang F. (2019). Photodynamic therapy guidelines for the management of oral leucoplakia. Int. J. Oral Sci..

[12] Wang Y., Li W., He Z. (2024). Multichiral mesoporous Silica screws with chiral differential mucus penetration and mucosal adhesion for oral drug delivery. ACS Nano.

[13] Wang X., Yuan Z., Tao A. (2022). Hydrogel-based patient-friendly photodynamic therapy of oral potentially malignant disorders. Biomaterials.

[14] Tang Q., Song C., Wu X. (2024). Dual-functional core–shell microneedle patches for oral ulcers treatment. Chem. Eng. J..

[15] Sang Z., Zhu T., Qu X. (2025). A hyaluronic acid-based dissolving microneedle patch loaded with 5-aminolevulinic acid for improved oral leukoplakia treatment. Colloids Surf. B Biointerfaces.

[16] Qin W., Ma Z., Bai G. (2025). Neurovascularization inhibiting dual responsive hydrogel for alleviating the progression of osteoarthritis. Nat. Commun..

[17] Wang Y., Guo J., Cao X. (2023). Developing conductive hydrogels for biomedical applications. Smart Medicine.

[18] Su H., Chen Y., Xuan Z. (2025). Permeable hydrogel encapsulated Osteosarcoma-on-a-Chip for high-throughput multi-drugs screening. Smart Med.

[19] Liu C., Zhao Q., Cao Y. (2025). Bioinspired structural color hydrogel skin from nonclose-packed colloidal crystal arrays for epidermal sensing. ACS Appl. Mater. Interfaces.

[20] Song C., Liu R., Kong B. (2024). Functional hydrogels for treatment of dental caries. Biomed Technol..

[21] Bian S., Ye H., Wang P. (2025). Antibacterial hydrogel: the sniper of chronic wounds. BMEMat.

[22] Zhao C., Cai L., Nie M. (2021). Cheerios effect inspired microbubbles as suspended and adhered oral delivery systems. Adv. Sci. (Weinh.).

[23] Madl C.M., Wang Y.X., Holbrook C.A. (2024). Hydrogel biomaterials that stiffen and soften on demand reveal that skeletal muscle stem cells harbor a mechanical memory. Proc. Natl. Acad. Sci..

[24] Ding Z., Hu X., Liang W. (2025). Dual-functional guanosine-based hydrogel: high-efficiency protection in radiation-induced oral mucositis. J. Mater. Chem. B.

[25] Ahmad U., Hanaffi W.N.W., Islam A. (2025). Cutting edge strategies for diabetic wound care: nanotechnology, bioengineering, and beyond. BMEMat.

[26] Sheng Y.-J., Chen Y., Qiu J.-F. (2023). Adhesive hydrogels for bioelectronics. Biomed Eng Commun.

[27] Zhu Z.-R., Huang J.-N., Li J.-Z. (2024). Janus hydrogel/electrospun-membrane dressing enhancing wound healing in rats. Biomed Eng Commun.

[28] Song C., Wu X., Wei Z. (2024). Dental pulp stem cells-loaded kartogenin-modified hydrogel microspheres with chondrocyte differentiation property for cartilage repair. Chem. Eng. J..

[29] Song C., Liu R., Fang Y. (2024). Developing functional hydrogels for treatment of oral diseases. Smart Medicine.

[30] Zhao Y., Song S., Ren X. (2022). Supramolecular adhesive hydrogels for tissue engineering applications. Chem. Rev..

[31] Cao Y., Liu C., Ye W. (2025). Functional hydrogel interfaces for cartilage and bone regeneration. Adv. Healthcare Mater..

[32] Fan M., Yang J., Zhen L. (2025). A mussel-inspired wet-adhesive prolonged-acting antibacterial hydrogels for the treatment of periodontitis. Chem. Eng. J..

[33] Ding M., Zhang Y., Li X. (2024). Simultaneous biofilm disruption, bacterial killing, and inflammation elimination for wound treatment using silver embellished polydopamine nanoplatform. Small.

[34] Zhou M., Lin X., Wang L. (2023). Preparation and application of hemostatic hydrogels. Small.

[35] Zhang Y., Ye S., Zhou Y. (2024). Salvianolic acid B as a potent nano-agent for enhanced ALA-PDT of oral cancer and leukoplakia cells. Oral Dis..

[36] Li X., Lovell J.F., Yoon J. (2020). Clinical development and potential of photothermal and photodynamic therapies for cancer. Nat. Rev. Clin. Oncol..

[37] Guo Q., Ji X., Zhang L. (2024). Differences in the response of normal oral mucosa, oral leukoplakia, oral squamous cell carcinoma-derived mesenchymal stem cells, and epithelial cells to photodynamic therapy. J. Photochem. Photobiol., B.

[38] Zhang R., Gao T., Wang D. (2023). Photodynamic therapy (PDT) for oral leukoplakia: a systematic review and meta-analysis of single-arm studies examining efficacy and subgroup analyses. BMC Oral Health.

[39] Wang F., Song Y., Xu H. (2024). Prediction of the short-term efficacy and recurrence of photodynamic therapy in the treatment of oral leukoplakia based on deep learning. Photodiagnosis Photodyn. Ther..

[40] Lv Q., Zhang Y., Yang R. (2023). Photoacoustic imaging endometriosis lesions with nanoparticulate polydopamine as a contrast agent. Adv. Healthcare Mater..

[41] Cao Y. (2025). Human umbilical vein endothelial cells (HUVECs) in pharmacology and toxicology: a review. J. Appl. Toxicol..

[42] Siddiqui S.A., Siddiqui S., Hussain M.A.B. (2022). Clinical evaluation of a mobile, low-cost system for fluorescence guided photodynamic therapy of early oral cancer in India. Photodiagnosis Photodyn. Ther..

[43] Zhu S., Zhao B., Li M. (2023). Microenvironment responsive nanocomposite hydrogel with NIR photothermal therapy, vascularization and anti-inflammation for diabetic infected wound healing. Bioact. Mater..

