# Supplementary material for: Adhesive hydrogel delivering ALA prevents the malignant transformation of oral leukoplakia
Source: Mater Today Bio. 2026 Mar 10;37:103017. doi: 10.1016/j.mtbio.2026.103017 (PMC13000714; doi:10.1016/j.mtbio.2026.103017)
Supplement: Multimedia component 1 [file mmc1.docx]

Supporting information

**Adhesive hydrogel delivering ALA prevents the malignant transformation of oral leukoplakia**

*Lin Lin, Jianchuan Ran, Yan Zhang, Shilin Guo, Xiteng Yin, Chuanchao Tang, Yufeng Wang, Wei Han, Wenmei Wang, Chuanhui Song*

*Nanjing Stomatological Hospital, Affiliated Hospital of Medical School, Institute of Stomatology, Nanjing University, Nanjing 210008, China.*

Supplementary Table 1. Inclusion/exclusion criteria for clinical application of ALA-hydrogel DDS

| Inclusion criteria | Exclusion criteria |
| --- | --- |
| - Informed consent, voluntary principle - Age 18-65 years old, both sexes - Patients with oral leukoplakia confirmed by histopathological examination | - Age < 18 or > 65 - Combined with oral ulcer, burning mouth syndrome, gingivitis, periodontitis and other oral diseases - With open wounds in the mouth - Porphyria, coagulopathy, pregnant women, uncontrolled serious systemic diseases (such as hypertension, heart disease, diabetes, severe liver and kidney dysfunction, malignant tumors, etc.) - Allergic to light, porphyrins, anesthetics, or hydrogels |


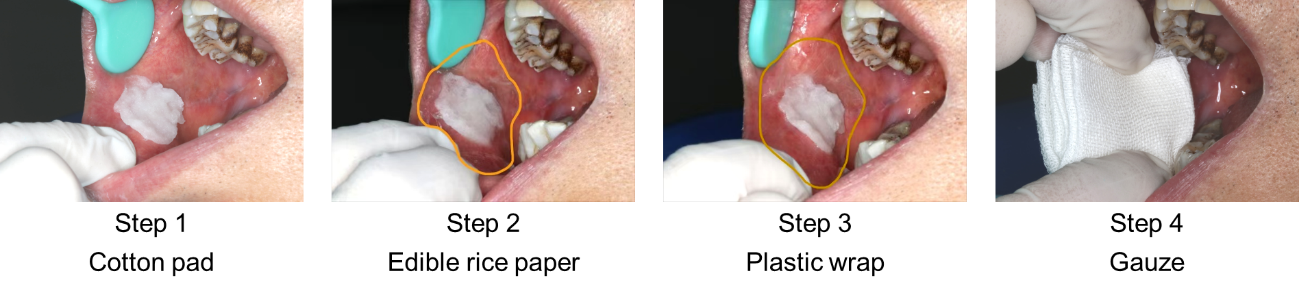


Figure. S1 Conventional administration of ALA-PDT.


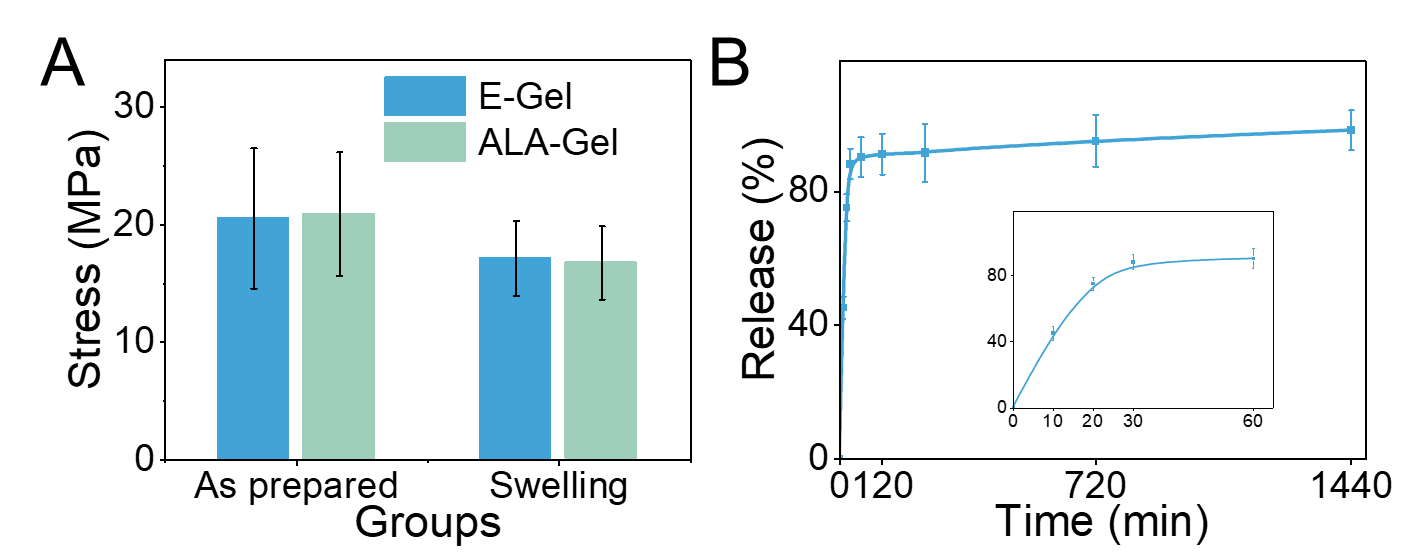


Figure. S2 (A) The stress effect before or after swelling of the hydrogel with or without ALA. (B) The ALA release profile of the ALA-Gel in the vertical Franz diffusion cell experiment.


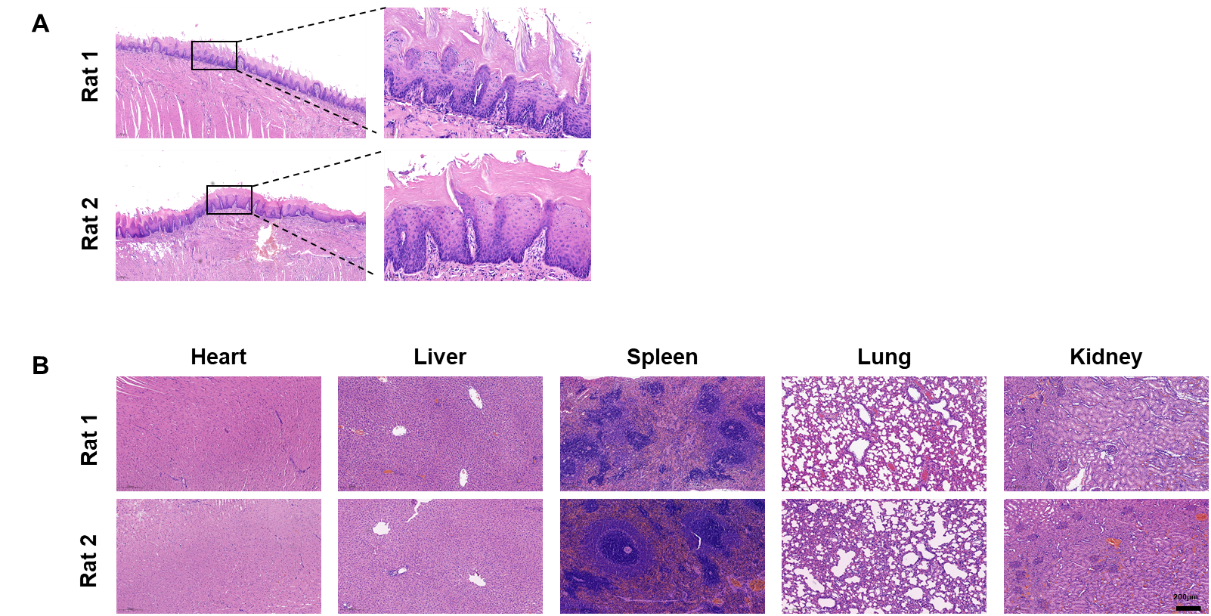


Figure. S3 H&E staining images of the tough (A) and the main organs (B) in rat1 and rat2. Scale bar: 200μm.


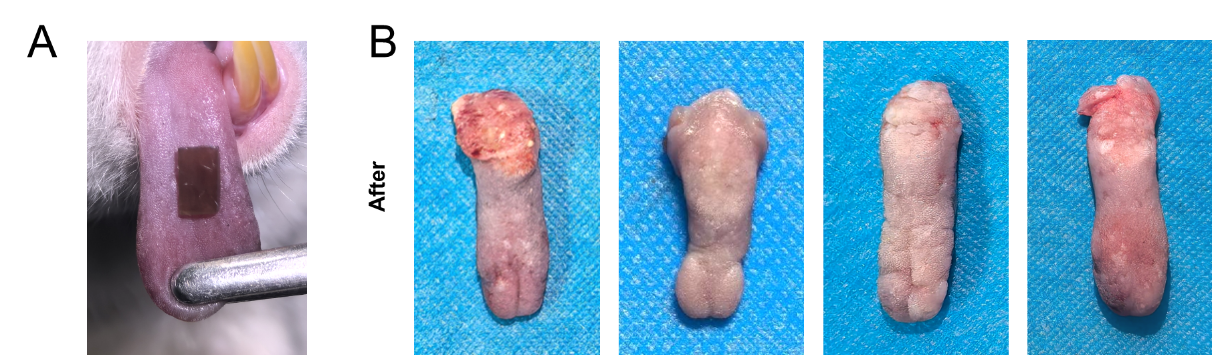


Figure. S4 (A) The application situation of the ALA-Gel in the rat oral site. (B) The digital photo of the tongue after the treatments.


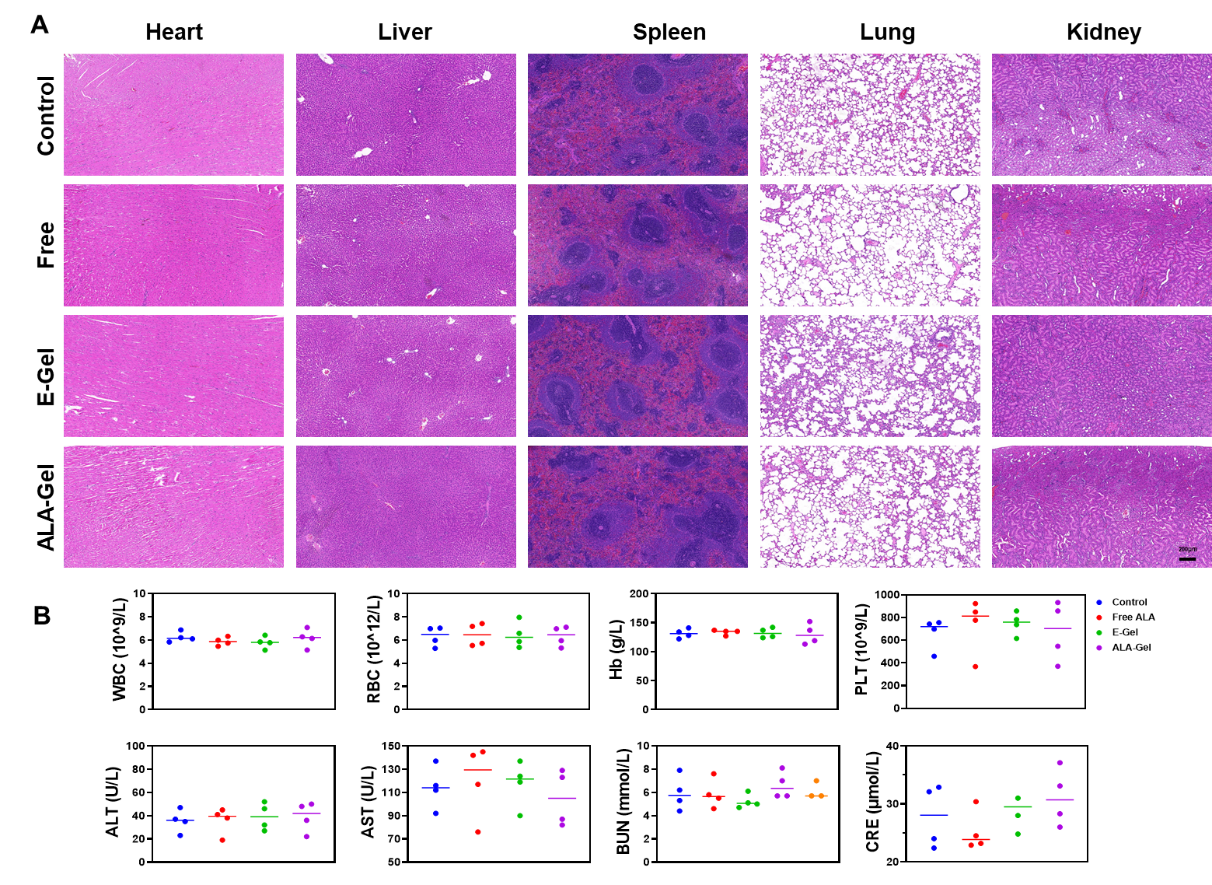


Figure. S5. (A) H&E staining images of the main organs in different groups of rats. Scale bar: 200 μm. (B) Blood samples were obtained from the eyes of Wistar rats. The blood routine, liver function, and kidney function indexes showed no differences among the groups, suggesting the safety of this therapy.
